# Professional Labels

**Published:** 1891-04-18

**Authors:** 


### The Doctor's Armchair.

### PROFESSIONAL LABELS.

When a man puts a label on himself he thereby proclaims to all men that he is conscious of his own obscurity. Labels have their uses; but to attach a label to an elephant or an oak tree, to a portrait of Lord Beaconsfield or of Mr. Gladstone, would be a stupid impertinence. The most obscure and least worthy of all tradesmen is generally he who, not content with putting his name over his shop door and his goods into the window, pushes a red deal board half-way across the footpath and prints on it in staring white letters—"This is Jones." The result of such a method of appeal to the public is that every man of sense and taste at once resolves that, so far as he is concerned, the advertiser shall continue to be "Jones" the unknown, and nothing else but "Jones" the unknown, to the end of time.

There is a certain class of medical men who are not content to be plain doctors and surgeons, but who prefer to be designated more precisely as "homœopaths," "allopaths," "hydropaths," "electropaths," and what not. One does not quite like to say that all such doctors are stupid fellows; still less does one wish to think that any of them are designing self advertisers of the unskilful sort. Nevertheless, everybody would admit that when a man is entitled by law to put up a door-plate which announces that he is "Dr. Robinson," or "Mr. Smith, Surgeon," he has quite sufficiently differentiated himself from other men for all practical purposes. There is no necessity whatever to put on the separatist label of "homœopath," "hydropath," "electropath," or the like. It is sufficient for the public to know that the doctor's business is to heal; the manner in which he will set about his work he decides for himself: and the public, at any rate, are no fit judges of its propriety or of its superiority to other methods of carrying on the business of healing. It is a common thing to put upon orthodox practitioners the label of "allopathy:" they are designated "allopaths." It is hardly necessary to say that no orthodox practitioner of any education ever calls himself an "allopath," or encourages anybody else to call him by such a name. The cognomen has no meaning at all in this particular connection. In fact the whole series of words ending in "pathy" are ridiculous from the point of view of elementary classical knowledge. According to the derivation of the words a "homœopath" is simply a "similar sufferer," and an "allopath" is "another sufferer," or a "different sufferer." If the terms have any application at all in medicine they apply to the patient, not to the doctor. It would be just as proper and as classically correct to call a physician a "bag-pipe" or a "jew's-harp" as to call him an "allopath." Any one of these three terms would be quite as descriptive as any other, because not one of them is descriptive at all.

There seems to be something in human nature which impels men to separate themselves into different schools of thought. We all discover, indeed, sooner or later, that we ourselves have special aptitudes for dealing with one thing, or one class of things, and that other men have special aptitudes for other classes of things. When we have attained to a little common sense and tolerance of spirit we see clearly that this is a good arrangement; and we never think of quarrelling with the butcher because he is not a doctor, or with the tailor, because he is not a captain in the Guards. But when we leave the arena of the great world and come into the specialised professions, we seem to suddenly lose our common sense and our spirit of tolerance. We insist that we ourselves see everything exactly as it is, and as it ought to be, and that if anybody else sees differently he is an ill conditioned person, who if he were to receive his due, would be whipped at the cart's tail, or have his ears cut off. Most people seem to think that it is the so-called "allopath" who is the grand inquisitor, and the homœopath who is the persecuted martyr. But the man who has been twenty years in medical practice has long ago exploded to his own mind that popular fallacy. To begin with, there is nothing in the constitution and rules of the medical cosmos to prevent a man from practising homœopathically to the end of time, if such be the bent of his mind. No orthodox practitioner will, on such a ground, refuse to meet him in consultation, or treat him in any other way than as a regular professional brother. It is true the orthodox man may have a private and inner conviction that his homœopathically inclined confrère is a "faddist," and possibly a fool. But he will not regard him as a cheat.

Why, then, does the orthodox doctor decline to meet in consultation the man who calls himself a "homœopath"? Simply because he does "call himself a homœopath." He puts a differentiating label upon himself; he declares that he is not a mere doctor who will treat his patients in any and every kind of way which their cases may require; but a separatist, a sectarian, a man of one idea, who will treat his patients in one particular way, and in no other. He classes himself, by his own free choice, with faith-healers, hypnotists, peculiar people, and the like. He is the sort of man who always insists upon travelling from London to St. Albans in a donkey cart, and he will not travel in any other way to save his life, or, for that matter, anybody else's. To him the railway train and the stage coach, and even the bicycle, are devices of the devil for putting obstructions and difficulties in the way of the only legitimate traveller—the driver of the donkey cart. The man who labels himself homœopath prefers to be singular; he invites other doctors to treat him with scorn; he is a martyr by preference and choice. Whether his motive be money, or love of singularity, or mere childishness of judgment it is not for us to determine. What, however, we do know is that any properly qualified medical man can practise the medical art in any way which his own honest judgment directs without incurring a shadow of suspicion or censure at the hands of his medical brethren. It is only when he himself has assumed a definite and conspicuous separatist label that he is regarded as a separatist. For this he has no one to thank but himself.

It is curious that we should have to go back to the New Testament for a lesson in scientific and medical unity. St. Paul says that in true Christianity there is "neither Greek nor Jew, Barbarian nor Scythian, bond nor free, but all are one in the life of faith." In medicine sectarianism is an absurdity, and an absurdity of the most childish kind. What is the business of the doctor? It is to heal! And how? By any and every method which he finds of service in his work. These are the two prime articles of the medical faith. Are they not wide enough to include every possible class of practitioner? He who cannot live under so broad and all-embracing a sky as this has no right to be surprised if he finds that men regard his restless vagaries with suspicion, and incline to think that if he is not a fool he is possibly something worse.

